# Application of the Milan System for Reporting Salivary Gland Cytology: A Prospective Study

**DOI:** 10.30699/IJP.2023.199632.3098

**Published:** 2023-10-15

**Authors:** Reema Bhushan, Jyoti Priyadarshini Shrivastava, Varsha Verma

**Affiliations:** *Department of Pathology, Gajra Raja Medical College, Madhya Pradesh, India*

**Keywords:** Fine-needle aspiration, Histopathology, Milan classification, Parotid gland, Salivary gland

## Abstract

**Background & Objective:**

The Milan system of classification of the salivary gland lesions came up with an aim to establish a universal reporting protocol. The aim of this study was to classify the fine-needle aspiration cytology (FNAC) cases of salivary gland according to the Milan system.

**Methods:**

All the cases presenting with salivary gland lesion for FNAC were considered. The clinical data was recorded. Cytology findings were analyzed according to the Milan System. Histopathological correlation was made wherever available.

**Results:**

A total of 100 cases of salivary gland lesions were collected and categorized according to the Milan system. They were correlated with histopathology in 45 cases. The patients’ age varied from 2-85 years. Parotid gland was the most commonly affected. Category 1 (non-diagnostic) comprised of three cases. Category 2 (non-neoplastic) had 40 cases. In category 4a (benign) there were 43 cases, and the most common lesion was pleomorphic adenoma. Category 5 (suspicious of malignancy) comprised of 3 cases. Category 6 (malignant) comprised of 11 cases and the most common lesion was mucoepidermoid carcinoma. In category 2, the cytological findings of 5 cases were concordant with histopathology while, 2 were discordant. In category 4a (benign), 20 cases were concordant, and 3 cases were discordant (2 cases were mucoepidermoid carcinoma, 1 was adenoid cystic carcinoma on histology). The risks of malignancy in NN, AUS, benign, SOM, and malignant were 33.3, 2.5, 0, 7, 66.6, and 100%, respectively.

**Conclusion:**

Milan system of reporting salivary gland cytopathology may have great potential of escalating clinical communication and may guide appropriate treatment.

## Introduction

Salivary gland tumors comprise approximately 3-5% of the head and neck tumors and 0.5% of all malignant tumors (1). Annual incidence rate of salivary gland tumors varies between 0.4-13.5 cases/100,000 individuals (2). The majority of these tumors are benign and only 20% are malignant (3). Tumors can occur in both major and minor salivary glands. Eighty percent of the major salivary gland tumors occur in the parotid gland. Most minor salivary gland tumors are located in palate (4). Fine-needle aspiration cytology (FNAC) is accepted by the surgeons as it is primary investigation for the evaluation of salivary gland lesions (5). FNA is always a favorable method for the diagnosis because incisional or core needle biopsy is a much invasive procedure with a tendency to be a potential source of infection (6). It provides a minimally invasive, safe, cost effective, and useful tool in identifying salivary gland nodules as benign or malignant, thus reduce unnecessary invasive surgical procedure in the patients with benign diseases (6-9). The diagnostic sensitivity of FNAC of the salivary gland neoplasms ranges from 62-97%, the specificity ranges from 80-100% and the accuracy from 86-98% (10, 11, 12). The cytological evaluation of salivary gland tumors has drawbacks because of wide range and heterogeneous nature of the benign and malignant tumors and overlapping cytological features, thus making the diagnosis problematic. Other studies stated that FNAC in salivary gland lesions is with pitfalls and the reason is for being multifactorial (13, 14, 15). 

Current reporting system in salivary gland cytology incorporates a wide diversity of diagnostic categories and descriptive reporting without any definite conclusion on the malignancy risk. There is very well-established reporting by Bethesda system for the cervical and thyroid cytology. To bring uniformity in reporting of lesions in salivary gland, international experts in salivary gland cytopathology proposed Milan system in 2015, a risk-based stratification system similar to the Bethesda system. The Milan System for Reporting Salivary Gland Cytopathology (MSRSGC) is a six-tier classification providing the standardized terminology and Risk of Malignancy (ROM) for each category, thus avoiding ambiguity often seen in FNAC interpretation and recommend clinical management (6). The present study is undertaken to know the incidence of salivary gland lesions especially with reference to malignancy in and around Gwalior region. The wide heterogeneity and similar cellular constituents shared by the salivary gland tumors result in diagnostic dilemmas especially in the basaloid tumors, oncocytic lesions, and mucous containing cystic neoplasms. The unified staging and risk stratification are required to bring the uniformity in reporting. International experts proposed the Milan system for reporting cervical and thyroid cytology (16). The Milan system of classification of the salivary gland lesions was sponsored by The American Society of Cytopathology and International Academy of Cytology. It is a user-friendly and internationally accepted, standard and uniform reporting system providing universal reporting protocol and better understanding of the lesions in relation to their clinical management.

Milan system for reporting salivary gland cytopathology consists of six diagnostic tiers:

Category 1: Non-diagnostic (ND): Material aspirated is insufficient for the cytological diagnosis. There are some exceptions, which include myxoid material and mucinous material.

Category 2: Non-neoplastic (NN): The lesions included are benign in nature in this category associated with reactive response to the inflammatory process such as acute or chronic sialadenitis, reactive lymph node, granulomatous sialadenitis, and infection. 

Category 3: Atypia of undetermined significance (AUS): Sample aspirated from lesions are indefinite for the diagnosis of neoplastic condition; but still a neoplasm cannot be excluded. The samples classified as “AUS” will usually show morphological overlap between non-neoplastic and neoplastic processes. 

Category 4a: Neoplasm A: The FNA specimen shows characteristic cytomorphological features of the selected benign epithelial or mesenchymal neoplasm. This include pleomorphic adenoma, Warthin’s tumor, lipoma, etc.

Category 4b: Neoplasm B: Salivary gland neoplasm of uncertain malignant potential (SUMP)-FNA aspirates that is determined of the neoplastic process, but cytologic findings cannot effectively distinguish between a benign and malignant tumor. This category should be used for the cases where a malignant neoplasm cannot be excluded. Cases will include cellular benign neoplasms, neoplasms with atypical features, and low-grade carcinomas. 

Category 5: Suspicious for malignancy: In this category, the FNA samples show cytological features that are highly suggestive of but doubtful for malignant, not all criteria for the selected diagnosis of malignancy are present. 

Category 6: Malignant: In this category, the FNA specimens are diagnosed as malignant.

The Milan system for reporting salivary gland cytopathology implicit the malignancy risk and suggest the necessary clinical management (17).

## Material and Methods

The study was initiated after obtaining the clearance from the Institutional Research and Ethics Committee. All the lesions of salivary glands belonging to all age groups were received in the Department of Cytology, Gajra Raja Medical College, Gwalior, during the period of 1st January 2021 till 31st June 2022. The patients’ age, sex, clinical history, and anatomical site were recorded. The recurrent cases and non-cooperative patients were excluded from the study. The patients’ consent was taken before the procedure. The FNA of the salivary gland swelling was performed under the strict aseptic condition. All the cases had palpable salivary gland lesions. All the FNAs were performed by the palpation. The procedure was done with the patient either in a supine or sitting position. The 23G needles and 10 cc syringes were used. In the cystic nodules, the cyst content was aspirated, centrifuged, and the slides were made from the sediment for the microscopic examination. Two air-dried slides were stained with LeishmanGiemsa stain. Two alcohol-fixed slides (95% ethyl alcohol) were stained with papanicolaou and examined under the light microscope. Provisional diagnosis was given. Cytology findings were analyzed according to the Milan system of the classification of salivary gland cytopathology into following diagnostic categories: Non-diagnostic (ND), Non-neoplastic (NN), AUS, Neoplasm A and B (SNUMP), Suspicious for malignancy, and Malignant. The preoperative cytological findings were correlated with the histopathological findings of surgically resected specimens whenever available and possible. The risk of malignancy for each cytological category was calculated based on the malignant histopathological diagnosis divided by the total number of cases in the corresponding category. The overall diagnostic accuracy, the sensitivity, specificity, positive predictive value, and negative predictive value were calculated by comparison with the histopathological diagnosis.

## Results

In the study period, 100 cases of salivary gland lesions were collected and categorized according to the Milan system. They were correlated with histopathologic results, which were available in 45 cases. The age of the patients in the present study ranged from 2-85 years. Maximum number of cases occurred in the age group of 41-50 years (n=32; 32%) followed by the age group of 31-40 years (n=16; 16%). Male to female ratio was 1.6:1. There were 13 cases of the patients less than 21 year of age (pediatric cases). The major salivary glands were affected more than minor salivary glands. The most commonly affected major salivary gland was the parotid gland (n=65; 65%), followed by the submandibular gland (n=34; 34%) and the least affected were the minor salivary glands (n=1; 1%). On FNAC, the samples were adequate in 97 cases and inadequate in 3 cases.

The FNA diagnosis of salivary gland lesions were categorized according to the Milan system. There were three cases in category 1. In category 2 (non-neoplastic), there were 40 cases including sialadenosis, acute and chronic sialadenitis, granulomatous sialadenitis, and acute nonspecific parotitis. The most common lesion was chronic sialadenitis (17/40). There was no case in category 3 (AUS). In category 4a (benign) there were 43 cases, and the most common lesion was pleomorphic adenoma (37/43). The 4b (SUMP) had no case. In category 5 (SOM), there were 3 cases with moderate to high grade nuclear features with overall cytomorphological features suggestive of malignancy, but not all the criteria for a specific diagnosis of malignancy were present. In category 6 (malignant), there were11 cases and the most common lesion was mucoepidermoid carcinoma (6/11) (Table 1). The histopathological follow-up was available in 45 cases (45%). 

**Table 1:** Categorization of the Cases According to Milan System

MILAN CATEGORY	CYTODIAGNOSIS	NUMBER OF CASES
Non-diagnostic	Cyst fluid (3)	3
Non-neoplastic	Acute non-specific parotitis (1) Granulomatous sialadenitis (3) Sialadenosis (9) Acute sialadenitis (10) Chronic sialadenitis (17)	40
3. AUS	-	-
Neoplasm 4a. Benign 4b. SUMP	Basal cell adenoma (1) Monomorphic adenoma (2) Warthin tumor (3) Pleomorphic adenoma (37)	43
5. Suspicious of Malignancy	-	3
6. Malignant	Mucoepidermoid/Epithelial-Myoepithelial (1) Adenocarcinoma NOS (1) Mucoepidermoid/Squamous cell carcinoma (2) Mucoepidermoid carcinoma (6) Mucoepidermoid/Adenocarcinoma(1)	11

**Fig. 1:**
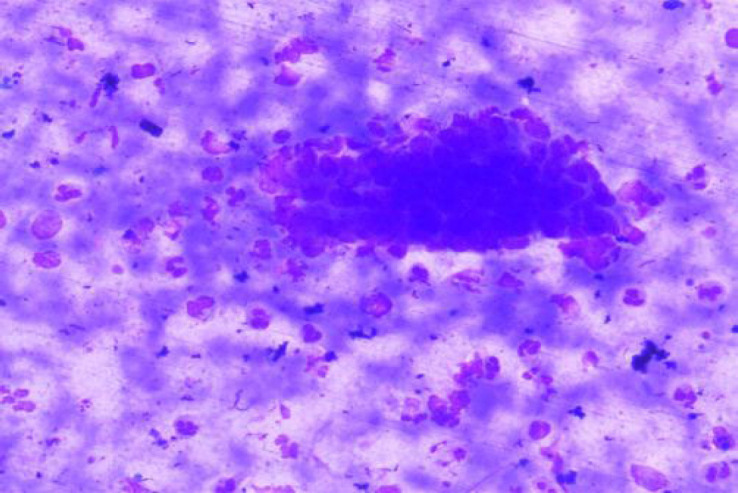
MGG Stain: Chronic Sialadenitis (100x)

**Fig. 2:**
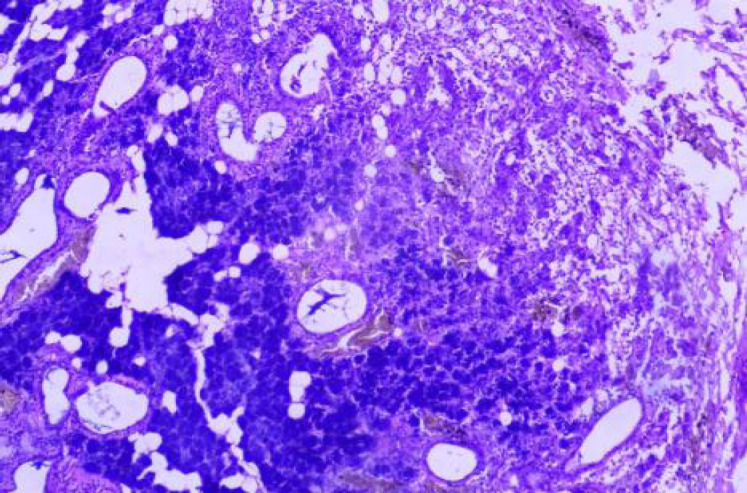
H and E Stain: Chronic Sialadenitis (100x)

According to the Milan system, the maximum number of cases were categorized as benign (n=43, 43%) with pleomorphic adenoma being the most common entity 37 cases followed by non-neoplastic lesion (n=40, 40%) with chronic sialadenitis (Figures 1 and 2) being the most common entity followed by the acute sialadenitis then sialadenosis followed by the malignant lesions (n=11; 11%) with maximum number of cases of mucoepidermoid carcinoma (n=7) (Table 2).

**Fig. 3:**
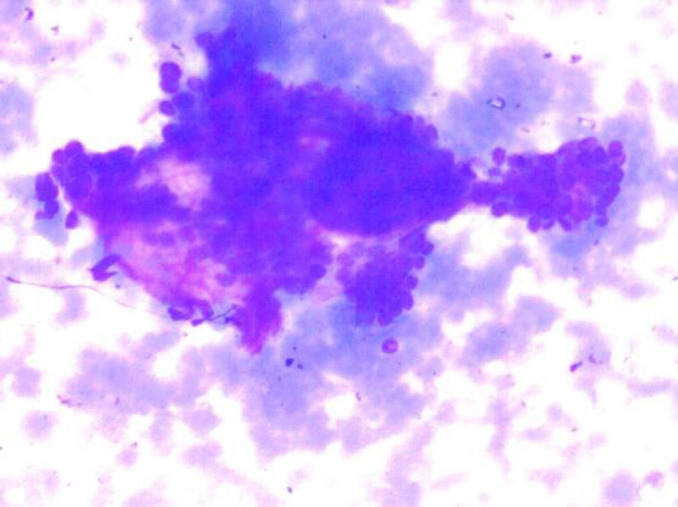
MGG Stain: Pleomorphic Adenoma (100x)

**Fig. 4:**
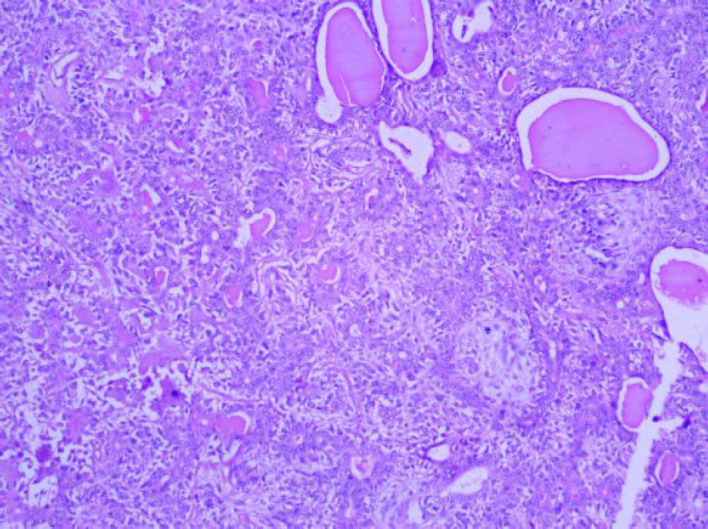
H and E Stain: Pleomorphic Adenoma (100x)

On cytology, there were 57 neoplastic lesions (57; 58.16%) of which 43 (75.4%) were benign, 11 (19.2%) were malignant and 3 (5.4%) were suspicious of malignancy. Benign lesions were more frequently seen in males more commonly in more than 40 years of age. Malignant and suspicious of malignancy lesions were predominantly seen in males, with all the cases seen in more than 40 year of age. The parotid gland (89.5%) was the most commonly involved by neoplasms, followed by submandibular gland (8.7%) and minor salivary gland (1.8%). Most of the cases of all salivary gland lesions were seen in the patients older than 40 years. Pleomorphic adenoma was the most common benign neoplasm more frequently seen in males, most commonly involving the parotid gland and the patients more than 40 years (Figures 3 and 4), while Warthin’s tumor was exclusively seen in the parotid gland and more than 40 years of age. Mucoepidermoid carcinoma was seen in males more than 40 years of age predominantly (Table 3).

On cytology, benign lesions (43; 43%) were more common in more than 40 years age group (25; 42.4%). Malignant lesions (11; 11%) were seen only in more than 40 years age group (11; 18.6%) and there was no case seen in less than 40 years (0; 0%). All non-diagnostic (3; 3%) cases were present in more than 40 years age group (3; 5.1%). Non-neoplastic lesions (40; 40%) were more commonly seen in less than 40 years age group (23; 56.1%). All cases of suspicious for malignancy (3; 3%) were seen in more than 40 years age group (3; 5.1%).

**Table 2:** Distribution of the cases on cytology and histopathology

S. No.	On Cytology	Number of cases	Follow up	Diagnosis on Histology
1.	Non-diagnostic	3	2	1-Chronic sialadenitis 1-Mucoepidermoid carcinoma
2.	Acute non-specific parotitis	1	-	-
3.	Granulomatous sialadenitis	3		
4.	Sialadenosis	9	-	-
5.	Acute sialadenitis	10	-	-
6.	Chronic sialadenitis	17	7	1-Warthin tumor 1-Low grade MEC 5-Chronic sialadenitis
7.	Basal cell adenoma	1	-	-
8.	Monomorphic adenoma	2	-	-
9.	Warthin tumor	3	2	1 Low grade MEC 1 Warthin tumor
10.	Pleomorphic adenoma	37	21	19- Pleomorphic adenoma 1-Adenoid cystic carcinoma 1-MEC
11.	Suspicious of malignancy	3	2	1-Adenoid cystic carcinoma 1-Acinic cell carcinoma
12.	Mucoepidermoid/Epithelial-Myoepithelial	1	1	High grade MEC
13.	Adenocarcinoma NOS	1	1	Adenoid cystic carcinoma
14.	Mucoepidermoid/Squamous cell carcinoma	2	2	2 Squamous cell carcinoma metastasis
15.	Mucoepidermoid carcinoma	6	6	6 Low grade MEC
16.	Mucoepidermoid/Adenocarcinoma	1	1	Adenocarcinoma metastasis

**Table 3:** Distribution of the salivary gland tumors according to the gender and age in 57 cases

	Male	Female	Total	Age		
TUMOR TYPE				<40Years(%)	>40years(%)	TOTAL
Benign	27 (71%)	16 (84.3%)	43 (75.4%)	18(100%)	25(64.1%)	43(75.4%)
Malignant	9 (23.6%)	2 (10.5%)	11 (19.2%)	0 (0%)	11(28.2%)	11(19.2%)
Suspicious of Malignancy	2(5.4%)	1 (5.2%)	3 (5.4%)	0 (0%)	3 (7.7%)	3(5.4%)
Total	38 (100)	19 (100%)	57 (100%)	18(100%)	39(100%)	57(100%)
LESION SITE						
Parotid	34 (89.5%)	17 (89.5%)	51(89.5%)	18(100%)	33(84.6%)	51(89.5%)
Submandibular	3 (7.9%)	2 (10.5%)	5(8.7%)	0 (0%)	5(12.84%)	5(8.8%)
Minor salivary gland	1 (2.6%)	0 (0%)	1 (1.8%)	0(0%)	19(2.56%)	1(1.7%)
Total	38 (100)	19 ( 100)	57 (100)	18(100%)	39(100%)	57(100%)
CYTOLOGICAL DIAGNOSIS						
Basal cell adenoma	1(2.63%)	0(0%)	1(1.75%)	0(0%)	1(2.56%)	1(1.75%)
Monomorphic adenoma	1 (2.63%)	1(5.26%)	2(3.5%)	0(0%)	2(5.12%)	2(3.5%)
Warthin tumor	2(5.26%)	1(5.26%)	3(5.26%)	0(0%)	3(7.7%)	3(5.26%)
Pleomorphic adenoma	23 (60.5%)	14(73.7%)	37(65%)	18(100%)	19(48.7%)	37(65%)
Suspicious of malignancy	2(5.26%)	1(5.26%)	3(5.3%)	0 (0%)	3(7.8)	3(5.2%)
Mucoepidermoid/Epithelial-Myoepithelial	1(2.63%)	0(0%)	1(1.7%)	0(0%)	1 (2.56%)	1(1.7%)
Adenocarcinoma NOS	0(0%)	1(5.26%)	1(1.75%)	0(0%)	1(2.56%)	1(1.75%)
Mucoepidermoid/Squamous cell carcinoma	1(2.63%)	1(5.26%)	2(3.5%)	0 (0%)	2(5.12%)	2(3.5%)
Mucoepidermoid carcinoma	4 (10.5%)	0(0%)	4(7%)	0 (0%)	4 (10.2%)	4(7%)
Mucoepidermoid/Adenocarcinoma	1(2.63%)	0(0%)	1(1.75%)	0(0%)	1(2.56%)	1((1.75%)
Mucoepidermoid/Epidermoid	2 (5.26%)	0(0%)	2(3.5%)	0(0%)	2(5.12%)	2(3.5%)
Total	38 (100)	19(100%)	57(100%)	18(18%)	39(100%)	57(100%)

**Fig. 5:**
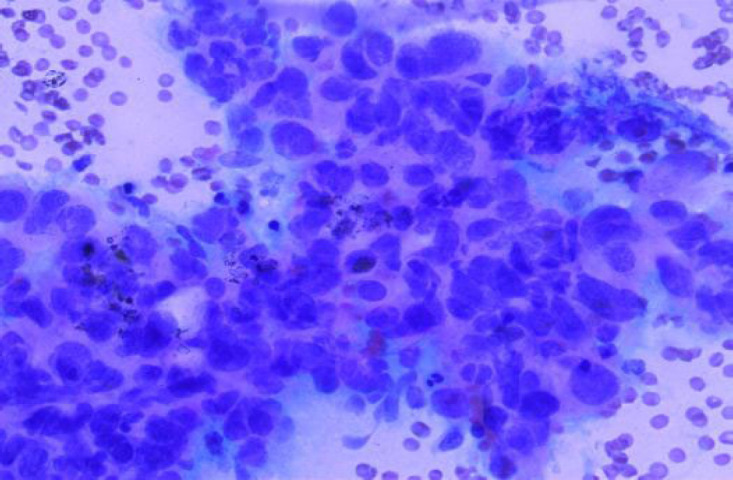
MGG Stain: Mucoepidermoid Carcinoma (400x)

**Fig. 6:**
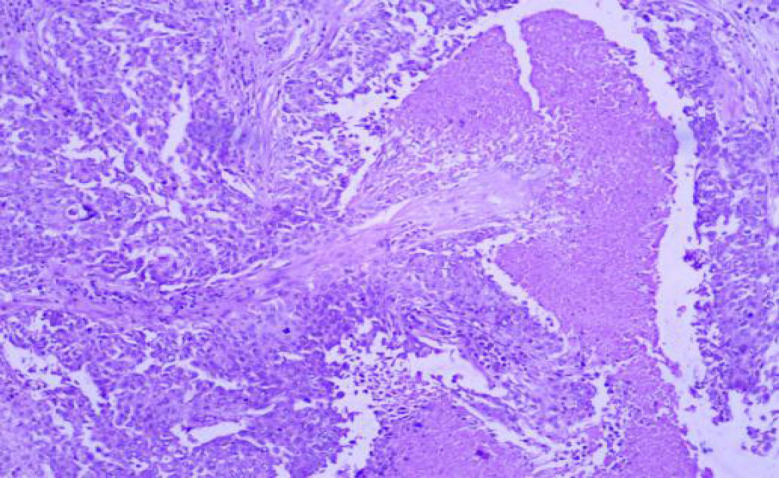
H and E Stain: Mucoepidermoid Carcinoma (100x)

**Fig. 7:**
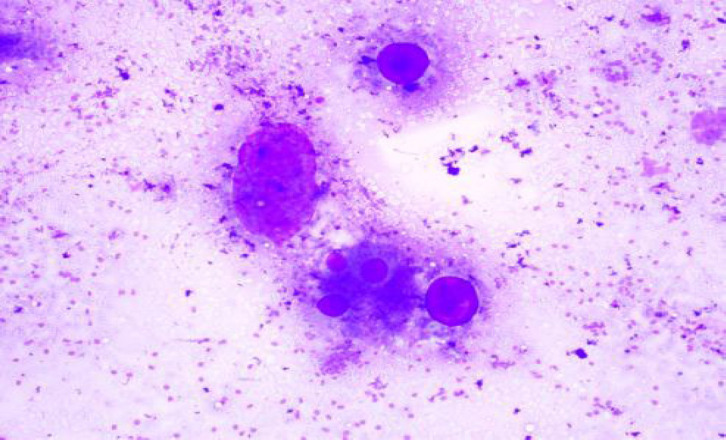
MGG Stain: Adenoid Cystic Carcinoma (100x)

**Fig. 8:**
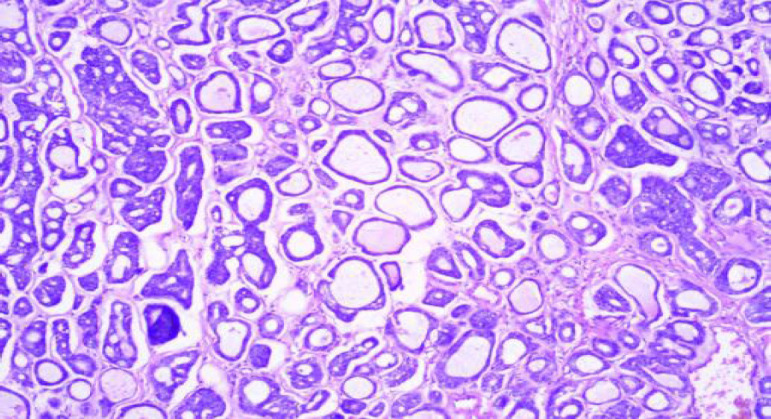
H and E Stain: Adenoid Cystic Carcinoma (100x)

After categorization of the salivary gland lesions according to the MSRSGC, cytohistological correlation was done with available follow-up information in 45 cases and further divided in concordant and discordant cases. Category 1 (ND): Cyst fluid, necrotic debris aspirate with few epithelial cells on cytology, which on histopathology turned out to be chronic sialadenitis (1), and mucoepidermoid carcinoma (1). Category 2 (NN): Out of 40 cases were in this category, histopathology of 7 cases was available. Cytological findings in 5 cases were concordant (chronic sialadenitis), 2 were discordant (1 was Warthin’s tumor, 1 was low grade MEC). Category 3 (AUS): There was no case in this category. Category 4a (benign): Out of 43 cases of cytology, follow-up of 23 cases (23/43, 53.5%) on histopathology was available, 20 cases were concordant (19 were pleomorphic adenoma and 1 was Warthin’s tumor), 3 cases were discordant (2 cases were mucoepidermoid carcinoma (Figures 5 and 6), 1 was adenoid cystic carcinoma: Figures 7 and 8) with our cytological findings. Category 4b: There was no case in this category. Category 5 (SOM): We had 3 cases in this category out of which 2 cases had histopathology available, which were concordant with our cytological findings with specific diagnosis of adenoid cystic carcinoma, and acinic cell carcinoma on histology. Category 6 (malignant): We had total of 11 cases on cytology. Histopathology was available of all cases (11/11, 100%) and all were concordant with our cytological findings, 7 cases of mucoepidermoid carcinoma, which on histopathology turned out to be MEC, 1 case of adenocarcinoma NOS /mucoepidermoid, which on histology turned out to be adenoid cystic carcinoma, 1 case of adenocarcinoma/mucoepidermoid carcinoma on cytology turned out adenocarcinoma metastasis, and 2 cases of squamous cell carcinoma/mucoepidermoid on cytology turned out to be squamous cell carcinoma metastasis. The risk of malignancy on comparing the Milan system with histopathology was ND as 33.3%, NN as 2.5%, benign as 7%, SOM as 66.6% and malignant as 100% (Tables 2 and 4).

In the present study, the sensitivity, specificity, positive predictive value, negative predictive value, and diagnostic accuracy of Milan system were 76.47%, 100%, 100%, 86.67%, 90.70%, respectively (Table 5).

**Table 4:** Cytohistological Correlation

Diagnostic Category	Number of the Cases on cytology	Follow up	Concordant Cases	Discordant Cases	Risk of Malignancy in Each Category
1.Non-diagnostic	3	2	-	1 Chronic sialadenitis 1 Low grade MEC	33.3%
2.Non-neoplastic	40	7	5	1Warthin tumor 1Low grade MEC	2.5%
3.AUS	-	-	-	-	0%
4.Benign Iva IVb	43	23	20	2 Mucoepidermoid carcinoma 1 Adenoid cystic carcinoma	7%
5.Suspicious of malignancy	3	2	1-Adenoid cystic carcinoma 1-Acinic cell carcinoma	-	66.6%
6.Malignant	11	11	7 MEC 1 Adenoid cystic carcinoma 1Adenocarcinoma metastasis 2 Squamous cell carcinoma metastasis	-	100%

**Table No. 5:** Sensitivity, specificity, PPV, NPV and accuracy of the MILAN system

Statistics	Value	Confidence interval (CI) 95%
Sensitivity	76.47%	50.10-93.19
Specificity	100%	86.77-100
Positive predictive value (PPV)	100%	-
Negative predictive value (NPV)	86.67%	73.40-93.87
Accuracy	90.70%	77.86-97.41

## Discussion

In the present study, 100 FNA of the salivary gland lesions were collected from January 2021 to June 2022 and categorized according to the MSRSGC. The percentages of cases in each category were 3, 40, 0, 43, 0, 3, and 11% in ND, NN, AUS, 4a benign and 4b SUMP, SOM and malignant, respectively. 

The range of age distribution in the present study was 2-85 years, the youngest and the oldest patients were 2-year-old male, and 85-year-old female, respectively. The range of age distribution in this study was closer to the studies done by Amita* et al. *(16), Mukundapai* et al. *(18), Gaikwad* et al. *(19), and Rohilla* et al. *(20). In the present study there was 97% adequate aspirates, which was similar to the studies done by Das* et al. *(21) and Nguansangiam* et al. *(22) with 96% and 94.8% adequate aspirates, respectively. In this study, males were more commonly affected than females with M:F ratio 1.6:1, which was similar to previous studies of Mukundapai* et al. *(18) , Rohilla* et al. *(20) , Geethalakshmi* et al. *(23), and Chandrakoshi* et al. *(24 ). Present study showed that parotid gland to be the most frequently involved site for the benign and malignant lesions followed by the submandibular gland and minor salivary gland similar to the study done by Mukundapai* et al. *(18).

Category 1: Non-diagnostic: There were 3 cases (3%), which were categorized in non-diagnostic category due to the presence of cyst fluid, necrotic debris, and insufficient to provide for any cytological diagnosis. The follow-up of two cases turned out to be chronic sialadenitis and mucoepidermoid carcinomas similar to the false negative case in the studies of Rohilla* et al. *(20), and Mishra* et al. *(25). The reason for this was the cystic nature of the neoplasm causing sampling error leading to inadequate aspirate. ROM in this category for the present study was 33.3%, which was in concordance with previous studies of Mukundapai* et al. *(18), Gaikwad *et al.* (19), Rohilla* et al. *(20), Chandrakoshi* et al. *(24), Mishra* et al. *(25), and Vishwanathan* et al. *(26). Non-diagnostic rate reported in the literature range from 5-10%. High non-diagnostic rate of 28.2% was reported in the study of Tahoun* et al. *(27), and Griffith* et al. *(28).

Category 2: Non-neoplastic: There were a total of 40 cases in this category from which sialadenitis was the most common lesion, which was similar to other studies performed by Jain* et al. *(7) and Das* et al. *(21). The follow-up was present in 7 cases and 5 cases were concordant with histological findings. ROM was 2.5%. In this study, non-neoplastic lesions were less than other reports. Rohilla* et al. *(20) reported higher number of non-neoplastic lesions (55.8%). Non-neoplastic lesions were more common in less than 40 years age group. 

There were 57 cases of neoplastic lesions, which were further divided into benign (43; 75.4%), malignant (11; 19.34%), and suspicious for malignancy (3; 5.26%). A study done by Nguansangiam* et al. *(22) reported 87.5% cases as benign, 8.3% cases as malignant, and 2.25% cases as suspicious for malignancy. The male:female ratios among benign and malignant were 1.68:1 and 4.5:1, respectively. Male predominance seen in the current study was similar to Rohilla* et al. *(20), Geethalakshmi* et al. *(23), and Chandrakoshi* et al. *(24) studies. However, study done by VK Sandhu* et al. *(18) reported female predominance in their study.

Category 4 (Neoplasm divided into 4a benign neoplasm and 4b SUMP): There were a total 43 cases, out of which, pleomorphic adenoma (37 cases) was the most common benign neoplasm, which was similar to the findings of VK Sandhu* et al. *(5), Amita* et al. *(16), Mukundapai* et al. *(18), Mishra* et al. *(25), and Viswanathan* et al. *(26). Histopathological correlation was available in 23 cases out of 43 cases, in which, 19 cases were concordant with cytological findings of the pleomorphic adenoma (PA) out of total 37 cases on cytology, but one case of PA turned out to be adenoid cystic carcinoma and one case turned out to be mucoepidermoid carcinoma similar to the pitfall, which was found in the study of Kotwal* et al. *(29). This diagnostic pitfall was due to varied appearance of the tumor (13). 

Another pitfall of FNAC is the difficulty to differentiate pleomorphic adenoma from lowgrade mucoepidermoid carcinoma. This pitfall can be attributed to the morphological similarities between pleomorphic adenoma and adenoid cystic carcinoma such as hyaline globules and myxoid stroma, which can lead to the misinterpretation in the diagnosis (30). There were three cases of Warthin’s tumor on cytology. Histopathology was available in two cases. One case was diagnosed as Warthin’s tumor on histopathology. Another one turned out to be mucoepidermoid carcinoma on histopathology similar to the study done by Mukundapai* et al. *(18) and Mishra* et al. *(25). The risk of malignancy in this category was found to be 7%. Allison DB in his study (31) found the ROM in the benign neoplasm category to be 3.0% and 1.3% for the pleomorphic adenoma and Warthin’s tumor, respectively. The ROM in the salivary gland neoplasm with uncertain malignant potential (SUMP) category was 2.7% and 18.8% for PA and WT, respectively (*P*= 0.0277). 

In the present study, all cases (37 cases) of pleomorphic adenoma were present in parotid gland more common in male than female. Overall non-neoplastic and benign neoplasms (83%) predominated over malignant (11%) lesions similar to the previous studies of Boccato* et al. *and Jayaram G* et al. *(32).

Category 5 (SOM): Total 3 (3%) cases were diagnosed under category 5 on cytology. Out of which, follow-up was available in two cases. One case was diagnosed as adenoid cystic carcinoma and the other as acinic cell carcinoma on histopathology. The risk of malignancy in the present study was 66.6% in this category, similar to the study done by Rossi* et al. *(16).

Category 6 (Malignant): Mucoepidermoid carcinoma was the most common malignant salivary gland neoplasm (63.6%) similar to the previous studies by Amita* et al. *(16) and Mukundapai* et al. *(18). The risk of malignancy was 100%, which was similar to the previous studies done by Rossi* et al. *(16), Rohilla* et al. *(20), and Abhilash* et al. *(33).

Among the pediatric and adolescent age group (<18 years), there were 14 cases, which presented palpable salivary gland swelling. Seven were in parotid gland and 7 were in submandibular region. Nine cases were given category non-neoplastic under the Milan system. There were five cases of benign category in the Milan system on cytology. Four were in parotid region and one in submandibular region. The histopathology was received in 4 cases. All turned out to be pleomorphic adenoma on histopathology. Maleki Z (34) performed a large international multi-institutional study focusing on the application of the Milan system in pediatric (<21 years) salivary gland lesions. In her study, the cases were categorized as non-diagnostic, 10.3%; non-neoplastic, 34.6%; AUS, 5.2%; benign neoplasm, 27.5%; SUMP, 7.5%; SM, 2.5%; and malignant, 12.4% cases. The ROMs after histology-cytology correlation was found to be non-diagnostic, 5.9%; non-neoplastic, 9.1%; AUS, 35.7%; benign neoplasm, 3.3%; SUMP, 31.8%; SM, 100%; and malignant, 100%. Mucoepidermoid carcinoma was the most common malignancy, and pleomorphic adenoma was the most common benign neoplasm in their study.

In the current research, the sensitivity, specificity, positive predictive value, negative predictive value, and diagnostic accuracy of the MSRSGC were 76.47%, 100%, 100%, 86.67% and 90.70%, respectively, which were close to those found in the study by Amita* et al. *(16), Rohilla* et al. *(20), Mishra* et al. *(25), Vishwanathan* et al. *(26), and Negi* et al. *(35). The ROM in the present study was category I-33.3%, category II-2.5%, category III-0%, category Iva-7%, IVb-0%, category V-66.6%, and category VI-100%, which were close to the studies done by Mukundapai* et al. *(18), Mishra* et al. *(25), and Modi* et al. *(36).

## Conclusion

Diagnostic challenges and wide heterogeneity in salivary gland tumors requires risk-based stratification scheme for the management of the patients. Histopathological diagnosis is the gold standard and decisive for the further management of the patients. However, the Milan system application in the present study showed consistent results compared to the worldwide study. Application of the Milan system of reporting salivary gland cytopathology (MSRSGC) has a great potential of escalating clinical communication i.e., to communicate the degree of suspicion of malignancy to the clinician (ROM), to guide treatment, provide counselling for the patients, and thus improving the overall management.

## Funding

 None.

## Conflict of Interest

None.
